# Spatial analysis of Mount St. Helens tephra leachate compositions: implications for future sampling strategies

**DOI:** 10.1007/s00445-015-0945-8

**Published:** 2015-06-13

**Authors:** P. M. Ayris, P. Delmelle, B. Pereira, E. C. Maters, D. E. Damby, A. J. Durant, D. B. Dingwell

**Affiliations:** Department of Earth and Environmental Sciences, Ludwig Maximilian University, Thereisenstrasse 41/III, 80333 Munich, Bavaria Germany; Earth and Life Institute, Université Catholique de Louvain, Croix du Sud 2, bte L7.05.10, 1348 Louvain-la-Neuve, Wallonia Belgium; Centre for Earth Evolution and Dynamics (CEED), University of Oslo, Blindern, Postbox 1028, 0315 Oslo, Norway; Geological and Mining Engineering and Sciences, Michigan Technological University, 1400 Townsend Drive, Houghton, MI 49931 USA

**Keywords:** Mount St. Helens, Tephra, Leaching

## Abstract

Tephra particles in physically and chemically evolving volcanic plumes and clouds carry soluble sulphate and halide salts to the Earth’s surface, ultimately depositing volcanogenic compounds into terrestrial or aquatic environments. Upon leaching of tephra in water, these salts dissolve rapidly. Previous studies have investigated the spatial and temporal variability of tephra leachate compositions during an eruption in order to gain insight into the mechanisms of gas-tephra interaction which emplace those salts. However, the leachate datasets analysed are typically small and may poorly represent the natural variability and complexity of tephra deposits. Here, we have conducted a retrospective analysis of published leachate analyses from the 18 May 1980 eruption of Mount St. Helens, Washington, analysing the spatial structure of the concentrations and relative abundances of soluble Ca, Cl, Na and S across the deposits. We have identified two spatial features: (1) concentrated tephra leachate compositions in blast deposits to the north of the volcano and (2) low S/Cl and Na/Cl ratios around the Washington-Idaho border. By reference to the bulk chemistry and granulometry of the deposit and to current knowledge of gas-tephra interactions, we suggest that the proximal enrichments are the product of pre-eruptive gas uptake during cryptodome emplacement. We speculate that the low S/Cl and Na/Cl ratios reflect a combination of compositional dependences on high-temperature SO_2_ uptake and preferential HCl uptake by hydrometeor-tephra aggregates, manifested in terrestrial deposits by tephra sedimentation and fallout patterns. However, despite our interrogation of the most exhaustive tephra leachate dataset available, it has become clear in this effort that more detailed insights into gas-tephra interaction mechanisms are prevented by the prevalent poor temporal and spatial representativeness of the collated data and the limited characterisation of the tephra deposits. Future leachate studies should aim to extensively sample across tephra deposit limits whilst simultaneously characterising deposit stratigraphy and tephra chemistry, mineralogy and granulometry, taking steps to ensure the quality and comparability of collected leachate datasets.

## Introduction

### Spatial analysis of tephra leachates

Tephra releases significant quantities of soluble elements upon leaching in water. These are generally accepted to originate from the dissolution of soluble sulphate and halide salts on particle surfaces (Óskarsson 1980; Rose et al. 1973; Rose 1977; Taylor and Stoiber 1973). These salts derive from several types of interactions between volcanic gases and tephra particles. Within the eruption plume, salts may be physically deposited onto tephra particles upon rapid cooling of magmatic gases in the atmosphere (Taylor and Stoiber 1973) or formed via chemical reactions between gases and tephra surfaces (Ayris et al. 2013, 2014; Hoshyaripour et al. 2014; Óskarsson 1980). In the cold volcanic cloud, salts may form on tephra surfaces due to dissolution of tephra by volcanic acid aerosols (Delmelle et al. 2007; Rose 1977). Both the eruption plume and volcanic cloud environments are subject to rapid changes in temperature (Mastin 2007), eruptive gas chemistry and redox state (Hoshyaripour et al. 2012), chemistry, pH and number concentration of aerosols (Herzog et al. 1998; Textor et al. 2003). Thus, each tephra particle carries a unique assemblage of salts imparted by its individual trajectory through a physically and chemically evolving eruption plume and volcanic cloud.

Leachate analyses measure the mean soluble salt concentrations on all tephra particle surfaces in deposit subsamples. However, the tephra particles found within those subsamples, irrespective of location within the deposit, are emplaced as a result of processes which dictate sedimentation and deposition of tephra particles into terrestrial environments (e.g. wind and bulk tephra particle properties; Bonadonna and Phillips 2003). In other words, the leachate composition from a tephra deposit subsample is the partial product of processes which are independent of those governing gas-tephra interactions, and hence, the leachate compositions may be variably decoupled from gas-tephra interaction processes. This fact leads to the realisation that it may only be possible to reassemble the syn- and post-eruptive chemical history of any deposit subsample by investigation of the spatial variability of leachate compositions from many such subsamples across the tephra deposit.

Tephra leachate studies have been undertaken for a number of purposes, from seeking mechanistic insight into volcanic processes to investigations of the environmental or health impacts of tephra leaching in receiving environments (Witham et al. 2005 and references therein). A number of these previous studies have investigated the spatial variability of tephra leachate compositions; some have used these data to infer changes in eruption dynamics and magmatic gas composition (Stoiber et al. 1981), under the assumption that relative and total concentrations of S, Cl and F in tephra leachates are a proxy for eruptive gas compositions, whereas others have analysed spatial trends in tephra leachate compositions to describe gas-tephra interaction processes in the eruption plume (Óskarsson 1980) or volcanic cloud (Bagnato et al. 2013; Rose 1977).

However, excluding the early work of Rose (1977), previous spatial studies (e.g. Armienta et al. 2002; Bagnato et al. 2013; Óskarsson 1980; Stoiber et al. 1981; Varekamp et al. 1984) have often relied on small datasets (i.e. 3–11 analyses). These may poorly represent the complex features of tephra deposits that may cover areas up to 10^2^–10^5^ km^2^. Although some studies have reported datasets comprising up to 34 leachate analyses, these are composites of much smaller datasets from different phases of prolonged eruptions spread over days or weeks (e.g. Chaitén, Durant et al. 2012; Eyjafjallajökull, Bagnato et al. 2013). Such datasets can also be limited in their capacity to represent the deposits of individual eruptive phases, particularly when emplaced under variable environmental conditions. It has thus proven difficult to demonstrate spatial variability in tephra leachate compositions from these datasets and, consequently, to unambiguously relate that variability to volcanic and/or depositional factors.

**Fig. 1:**
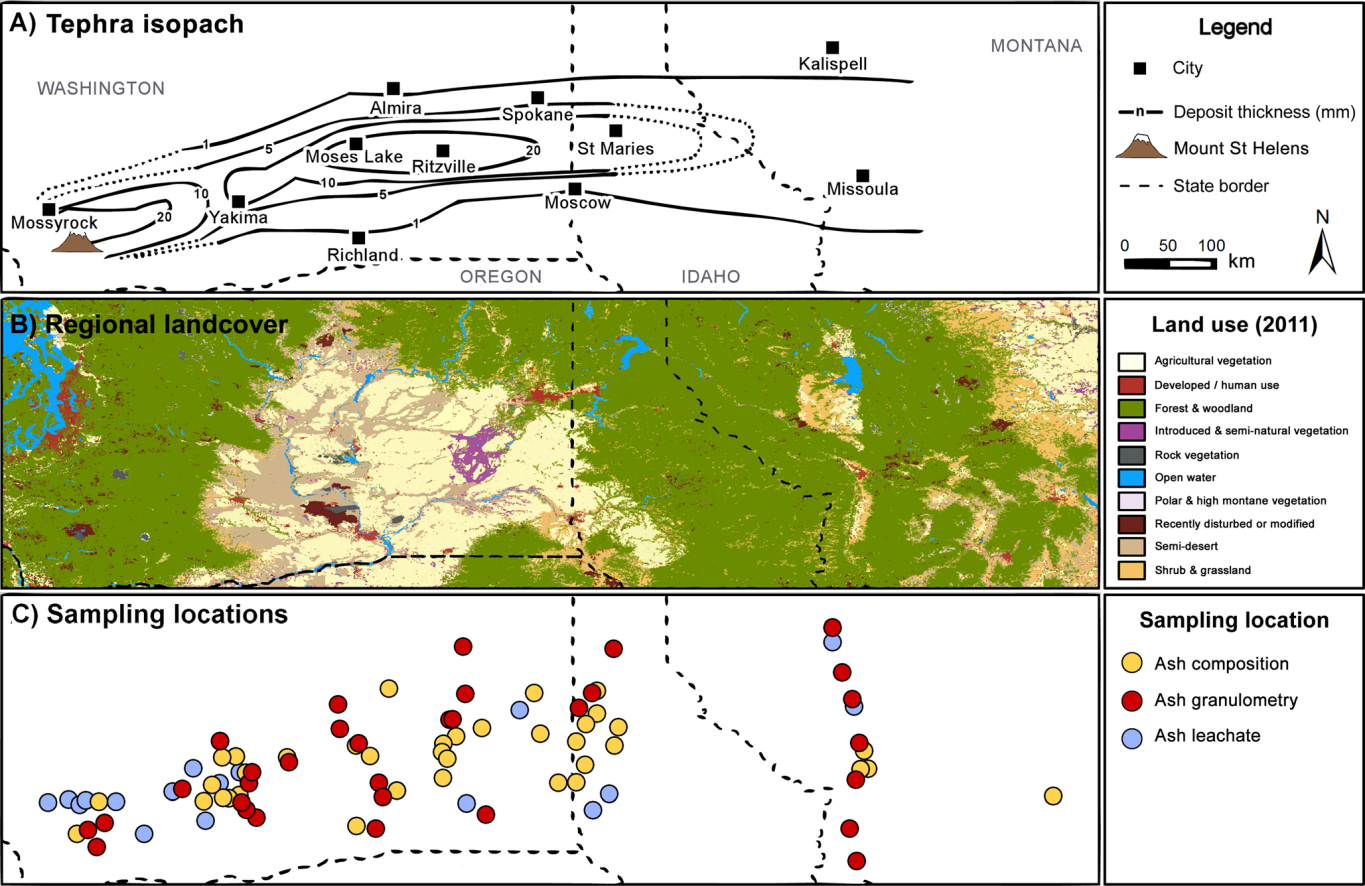
Maps of **a** MSH 18 May 1980 deposit, including isolines of total deposit thickness (mm) generated from data collated in Durant et al. (2009), and positions of state boundaries and cities named in the current study; **b** regional landcover for the year 2011, based on information from the ‘National Gap Analysis Program Land Cover Data—Version 2’; and **c** positions of locations sampled for tephra composition analysis (*yellow-filled circles*), granulometry measurements (*red-filled circles*) and tephra leachate analysis (*blue-filled circles*). Although the regional landcover map is based on data from 2011, it is sufficient to illustrate the broad extent of geographic areas in our discussion

Here we perform a retrospective evaluation of published leachate compositions from the 18 May 1980 eruption of Mount St. Helens (MSH), in Washington (WA), USA. The eruption datasets comprise more than 300 leachate analyses from 185 tephra samples, reported in ten different studies. Although, in our evaluation, differing leaching protocols render the majority of these incomparable (Table 1), the remaining comparable dataset still constitutes the largest for any single eruptive event in the published literature. Other tephra properties such as grain size distribution and bulk chemical composition have also been extensively documented (Fig. 1). The MSH eruption may thus represent the best opportunity for identifying and explaining any spatial variability in tephra leachate chemistry. We use statistical analyses to investigate spatial trends in total and relative abundances of soluble S, Cl, Na and Ca in tephra leachates. These trends are interpreted by reference to tephra deposit features and current understanding of gas-tephra interactions. Our analysis and interpretations emphasise the importance of extensive, rigorous sampling and in-depth characterisation of tephra deposits in leachate studies of future eruptions.

**Table 1:** List of papers (Fruchter et al. 1980; Hinkley et al. 1987; Jones and Gislason 2008; McKnight et al. 1981; Nehring and Johnston 1981; Smith et al. 1983; Sneva et al. 1982; Stoiber et al. 1981; Sung et al. 1982; Taylor and Lichte 1980) which report leachate compositions from the MSH eruption, detailing the number of samples collected (or collated) within the study; the number of leachate compositions reported by the study and the number of samples within that dataset which were extracted utilising only H_2_O; the number of leachate compositions discarded and the remaining viable data collated in our initial survey of the available studies. Additional information regarding the rationale for data exclusion are indicated below the table

	First author									
	Fruchter	Hinkley^a, b^	Jones^c^	McKnight^b^	Nehring^a^	Smith^b^	Sneva^a, b, d^	Stoiber	Sung	Taylor^e^
Year	1980	1987	2008	1981	1981	1983	1982	1981	1982	1980
Ash samples	9	69	1	1	13	19	33	21	3	16
Leachate compositions	9	143	1	1^a^	13	57	48	21	3	6
H_2_O-leach	9	68	1	0	13	14	19	21	3	6
Discarded compositions	0	38	1	0	4	0	14	0	1	0
Viable compositions	9	30	0	0	9	19	0	21	2	6

^a^Study reports unpristine ash

^b^Study reports values acquired from leaching experiments conducted in various leaching media

^c^Study reports leachate from a 28-year-old tephra sample, potentially influenced by ‘leachate decay’ (Jones and Gislason 2008)

^d^Study reports mean values from an unpublished dataset

^e^Study reports selection of values from an unpublished dataset

### MSH eruption overview

The MSH eruption consisted of six distinct phases of activity over the first 24 h of the eruption (Criswell 1987; Waitt and Dzurisin 1981; Sarna‐Wojcicki et al. 1981a; Carey et al. 1990; Pallister et al. 1992; see Table 1 in Durant et al. 2009 for a complete summary). The first phase began with the flank collapse landslide, the lateral blast resulting from the immediate depressurisation of the crystal-rich, dacitic cryptodome (Hoblitt et al. 1981) and subsequent co-ignimbrite plumes. The landslide and blast deposits were emplaced up to 12 km north of Mount St. Helens, between the volcano and the Mossyrock area in Washington (Fig. 1). Crystal-rich dacitic tephra from this initial phase was also deposited as fallout between MSH and Yakima, WA. The second phase featured a sustained Plinian column with a transition from the eruption of a dark-coloured dacitic tephra to a white, silica-rich dacitic pumice (Durant et al. 2009; Criswell 1987). Tephra was dispersed to the east of MSH; the dark-coloured tephra was deposited over Washington, only noted as a millimetre-thick basal layer at the WA-ID border by Sarna‐Wojcicki et al. (1981a). The third and fourth phases of the eruption were dominated by smaller explosive events with pyroclastic flows and associated co-ignimbrite column development (Durant et al. 2009). During these periods, fine-grained, glassy tephra was lofted into the atmosphere by co-ignimbrite plumes and dispersed over distal regions. The fifth and sixth phases of the eruption were dominated by small pyroclastic flows and weak tephra explosions (Criswell 1987). Changes in column height combined with vertical wind shear shifted the deposit axis approximately 40 km northwards throughout the period on activity on 18 May 1980 (Sarna‐Wojcicki et al. 1981a). The distal (100 s km from source) tephra deposit featured a mass deposition maximum centred over Ritzville, WA, which formed from fallout of loosely bound tephra aggregates (Durant et al. 2009).

## Methods

### Compiling a comparable leachate dataset

#### Comparability of different sampling and leaching methods

The 302 tephra leachate compositions collated from ten previous studies (Table 1) were extracted and analysed by a variety of sampling and analytical techniques, impeding cross-comparison. In our analysis, we do not consider any tephra previously exposed to rainfall, as the pre-analysis dissolution of soluble salts prevents comparison with pristine tephra (c.f. Hinkley et al. 1987). We also exclude tephra samples recovered during the eruption; 60 % of leachate compositions in Stoiber et al. (1981) and 9 % from Hinkley et al. (1987) are from specific times during the eruption. As the vast majority of leachate compositions in the wider dataset were recovered after tephra deposition had abated, it is difficult to draw any comparison between a small number of time-specific samples, representing the leachate chemistry of a single moment or period of the eruption, to samples which represent that of the whole eruption. Hinkley et al. (1987) also included a number of analytical duplicates of the same tephra samples; to avoid artificially inflating the collated leachate dataset, we replace those data with a single calculated mean for each duplicate pair.

Across the ten studies, most tephra leachate compositions were obtained using deionised or distilled water as the leaching solution. For the sake of comparison, studies that used other extractants such as H_2_SO_4_ and HNO_3_ (Smith et al. 1983) were discarded from the dataset. Analysis of a range of tephra/water ratios (1.6:1 to 1:14) and leaching times (1 to 25 h) was reported, further impeding comparison of data.

High tephra/water ratios can promote saturation of leaching solutions with respect to poorly and moderately water-soluble mineral phases, including gypsum (CaSO_4_⋅2H_2_O, solubility product *K*_Sp_ = 2.4 × 10^−5^) and fluorite (CaF_2_, *K*_Sp_ = 1.5 × 10^−10^; Barton 1991). Similarly, short leaching times may not achieve full dissolution of soluble salts. In both cases, leachate compositions may be unlikely to reliably reflect the soluble salt concentration on the tephra particle surfaces. In the case of the MSH leachate data, thermodynamic modelling and previous leaching studies (Taylor and Lichte 1980; Jones and Gislason 2008) imply that for H_2_O-leached tephra samples, there may be no significant influence of tephra/water ratio or leaching time. Mineral saturation indices for MSH leachates calculated using PHREEQC predicts that, of the various chloride and sulphate salts which may exist on tephra surfaces, only BaSO_4_ was saturated in leachate solutions. As neither Hinkley et al. (1987) nor Smith et al. (1983) reported significant increases in Ba in more dilute and/or acidic leachates from MSH tephra, saturation effects have little impact on our collated dataset.

IVHHN guidelines (Stewart et al. 2013) consider that a leaching time of 1 h is sufficient to extract a representative quantity of soluble salts from tephra surfaces. However, leaching in this time may not achieve total dissolution of the soluble salt load. During three successive 4-h leaching experiments on MSH tephra, Taylor and Lichte (1980) reported that the initial leach extracted 75 ± 20 % of the total soluble Ca, Cl, Na and S release. Similarly, in continuous leaching of MSH tephra within a plug flow-through reactor over 8 h, the data of Jones and Gislason (2008) show that an average of 54–68 (±18) % of the total release of Ca, Cl, Na and S was mobilised within the first 50–100 min. The comparable extraction in these two time periods implies that there may only be a small difference in tephra leachate extracted after 1 and 4 h of immersion. However, the contrast between these samples and those leached for 25 h may be more significant. For the purposes of this study, we do not exclude samples on the basis of leaching time, but the effect of prolonged leaching on our interpretation of the collated data is considered in the section “Validity of observed spatial trends”.

#### Elements of interest

**Fig. 2:**
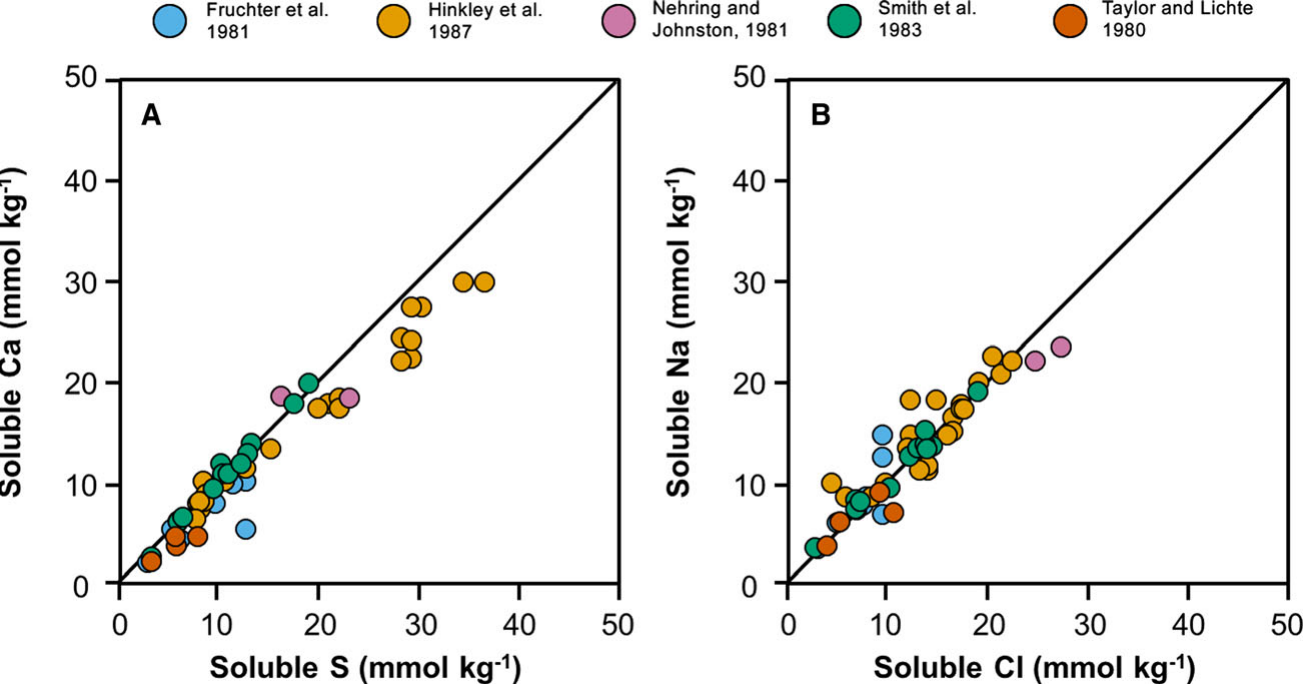
Concentrations of **a** soluble Ca vs soluble S and **b** soluble Na vs soluble Cl extracted from studies that report data for all four elements; *blue*, Fruchter et al. (1980); yellow, Hinkley et al. 1987; *pink*, Nehring and Johnston (1981); *green*, Smith et al. (1983); *orange*, Taylor and Lichte (1980)

The studies detailed in Table 1 collectively report concentrations for over 34 major, minor and trace elements in solution, with half of all analyses reporting non-zero concentrations of Ba, Ca, Cl, Cu, Co, F, Fe, Li, Mg, Mo, Na, S, Si, Sr, V and Zn. In all samples, the dominant constituents of leachate solutions are Ca, Cl, Na and S, and molar ratios of Na/Cl and Ca/S in MSH leachate solutions are approximately consistent with the 1:1 stoichiometric ratios expected from NaCl and CaSO_4_ dissolution (Fig. 2). Accordingly, we interpret trends in leachate compositions for MSH by reference to these four elements alone.

#### Exclusion of outliers

**Fig. 3:**
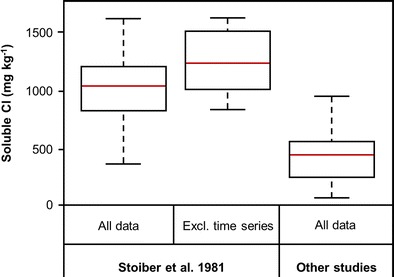
Boxplot comparison of all 21 soluble Cl data (mg kg^−1^) from Stoiber et al. (1981), the subset of six soluble Cl that are not attributed to a specific sampling time (e.g. Almira, 1800 hours) or period (e.g. Spokane, ‘early tephra fallout’), compared with all soluble Cl data from the five studies which also report Cl concentrations (Fruchter et al. 1980; Hinkley et al. 1987; Nehring and Johnston 1981; Smith et al. 1983; Taylor and Lichte 1980). The *red line* indicates the median of the respective datasets, whilst the extent of the *white bar* covers the interquartile ranges. The extent of the *dashed lines* indicates the maximum and minimum values of those datasets

We analyse variations in soluble Cl, Ca, Na and S concentrations to identify and exclude outliers. The concentrations reported by Stoiber et al. (1981) are consistently higher than those of all other studies (Fig. 3), despite overlap in the spatial distribution of tephra samples. The dataset of Stoiber et al. (1981) may either be subject to systematic analytical error or differences in sampling practices which are not documented in the original study; in either case, we exclude that dataset from our study.

We analyse the variation in leachate concentrations at locations where duplicate data from one or more studies were collected. At each sampling site, it was assumed that each measured value should depend upon (i) a ‘true’ concentration value at that point and (ii) a random error following a normal distribution of 0 mean and *σ*_D_ standard deviation. The ‘true’ concentration value at the point is estimated by the mean of the field duplicate data, and the standard deviation of the random error is estimated by the pooled standard deviation (Nič et al. 2009). The assumption of a normal distribution in random error within the data has been verified by a Shapiro-Wilk test, where *α* = 0.05. Statistical outliers in duplicate data were considered to be those which fell outside of a 95 % confidence interval calculated via Eq. (1).

1 $$ \overline{Y_i}\pm {\sigma}_{\mathrm{D}}*\mathrm{St} $$

where ${\overline{Y}}_i$ is the mean value of the field duplicate data at point *i*, *σ*_D_ is the pooled standard deviation and St is the 1-α/2 quantile of a t-distribution with the same degrees of freedom as the pooled standard deviation. We analysed S, Cl, Ca and Na and S/Cl, Na/Cl and Ca/S ratios following this treatment and excluded nine outliers from the collated elemental datasets: two from the soluble Ca dataset, two from soluble Cl, two from soluble Na and three from soluble S.

#### Leachate composition summary

The final collated MSH dataset utilised in our study comprises 56 leachate compositions from the MSH tephra deposit. This represents only 20 % of the original 302 sample dataset, but nevertheless remains the largest for any single studied eruption. Tephra leachates are distributed across 32 locations across the deposit, although these locations are reported with varying precision. For example, Hinkley et al. (1987) report sampling locations using latitude and longitude, accurate to two decimal places (±1 km), whilst Nehring and Johnston (1981) provided some locations utilising Public Land Survey System coordinates, introducing a maximum error of ±2 km. All other locations were reported with respect to a named city or landmark, sometimes combined with distance and bearing from that point (e.g. 20 km NW of Spokane). Those locations are presumed to be situated at, or oriented with respect to, the geographic midpoint of the named areas. Approximating the land area of the named locations to circular envelopes, errors based on the envelope radius are 0.2 to 7 km. For interpretation of broad-scale spatial features, there is little significance of these error envelopes; their areas are less than 0.5 % of the whole deposit.

### Additional tephra characterisation data

#### Grain size dependence of leachate compositions

As gas-tephra interaction mechanisms involve surficial reactions, leachate compositions should be normalised to the specific surface area (SSA, m^2^ g^−1^) of the tephra sample. No SSA analyses were undertaken in the collated studies; we therefore calculated geometric specific surface area (SSA_geo_) values, calculated via Eq. (2) (Rumstidt 2013), where *p*_d_ is particle diameter (m) and *ρ* is individual particle density (g cm^−3^), from 37 particle size distributions taken from across the MSH deposit (Durant et al. 2009).

2 $$ {\mathrm{SSA}}_{\mathrm{geo}}={\scriptscriptstyle \frac{6\times {10}^{-6}}{\rho {p}_{\mathrm{d}}}} $$

The particle size distributions of Durant et al. (2009) were measured using a Malvern Mastersizer 2000 laser diffraction analyser. We further analysed four more samples acquired for the current study using a Coulter Q-100 Laser Analyser and calculated their respective SSA_geo_ values. Both approaches assumed a particle refractive index of 1.6 and approximated particles to spheres with density of 2.5 g cm^−3^.

From this SSA_geo_ dataset, comprising 43 values in total, only three were located within the tephra fallout region west of Yakima, WA. Proximal deposits in this region are known to comprise material from multiple discrete events: the lateral blast, landslide and both co-ignimbrite and tephra fallout deposits (Waitt and Dzurisin 1981). As the complex, overlapping contributions of these disparate features cannot be represented by three samples alone, the three proximal SSA_geo_ values are excluded from our spatial analysis. The remaining 40 SSA_geo_ values were situated on a deposit map according to their stated sampling location, considering any apparent spatial trends evident in that map to reflect changes in deposit granulometry.

**Fig. 4:**
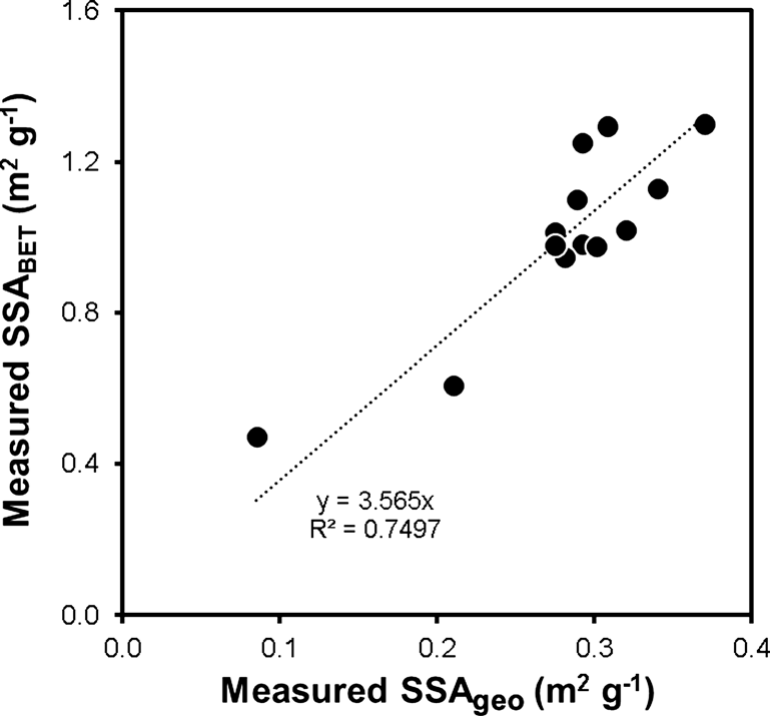
Comparison of SSA_geo_ calculated from measured tephra particle size distributions and the SSA_BET_ calculated from K_2_ adsorption isotherms for the same samples

A limitation of this treatment is that SSA_geo_ neglects the complex surface morphology of tephra particles, and so underestimates SSA (Ersoy et al. 2010). To determine whether such an impediment prevents interpretation of spatial trends in SSA_geo_, we determined the SSA of 13 tephra samples from our dataset, calculated by application of the Brunauer-Emmett-Teller (BET) theory (Brunauer et al. 1938) to measurements of Kr_2_ adsorption isotherms using a Micromeritics ASAP 2000 Surface Area Analyser. Figure 4 shows that for these samples, SSA measurements were approximately 3.5× higher than SSA_geo_, but that the two were positively correlated with *r*^2^ of 0.75. This implies that for illustrative discussion of relative trends in tephra SSA across the whole tephra deposit, the use of SSA_geo_ offers a reasonable approximation in this instance.

#### Bulk chemical composition of tephra

We collated 101 bulk chemical composition analyses for tephra samples recovered from 49 different locations from four different studies (Fruchter et al. 1980; Hinkley et al. 1987; Sarna‐Wojcicki et al. 1981b; Smith et al. 1983). The locations from which these data were obtained were described with similarly variable precision as those sampled for leachate analysis. For all locations identified by name only, we assign a latitude and longitude according to the method described in the section “Compiling a comparable leachate dataset”.

Hinkley et al. (1987) reported that for selected samples from across WA, ID and MT, leachate compositions showed significant correlation with bulk Ca, Na and Si content. We therefore focus our analysis of deposit bulk chemical composition on these three elements, expressed as oxides (wt%). We excluded four outliers: two for CaO, one for SiO_2_ and one for Na_2_O. The final collated dataset, averaging all replicate subsamples and excluding outliers, comprises 78 analyses. As multiple researchers often sampled in the same areas, these data were obtained from 44 discrete sampling locations.

### Data analysis

To investigate spatial trends in MSH tephra leachates, we plot all compositional data (i.e. leachate and bulk tephra analyses) according to its geographic position. Where field duplicate data exists, we calculate and plot the mean concentration or value for that location. As no field duplicate data were available for SSA_geo_, each value obtained was assumed to be representative of the tephra deposit at that location.

Based on the mean values for each location, we utilised the Geostatistical Analyst extension of the ArcGIS 10.1 software (ESRI 2012) to generate maps that illustrate any spatial structure in the distribution of leachate concentrations, tephra deposit bulk chemistry and granulometry across the deposit. These maps were generated by interpolation of data via ordinary kriging. Variograms were fitted using the ‘nugget effect’ and ‘spherical model’ structures.

## Results

### Spatial analysis of raw data

#### Leachate compositions

**Fig. 5:**
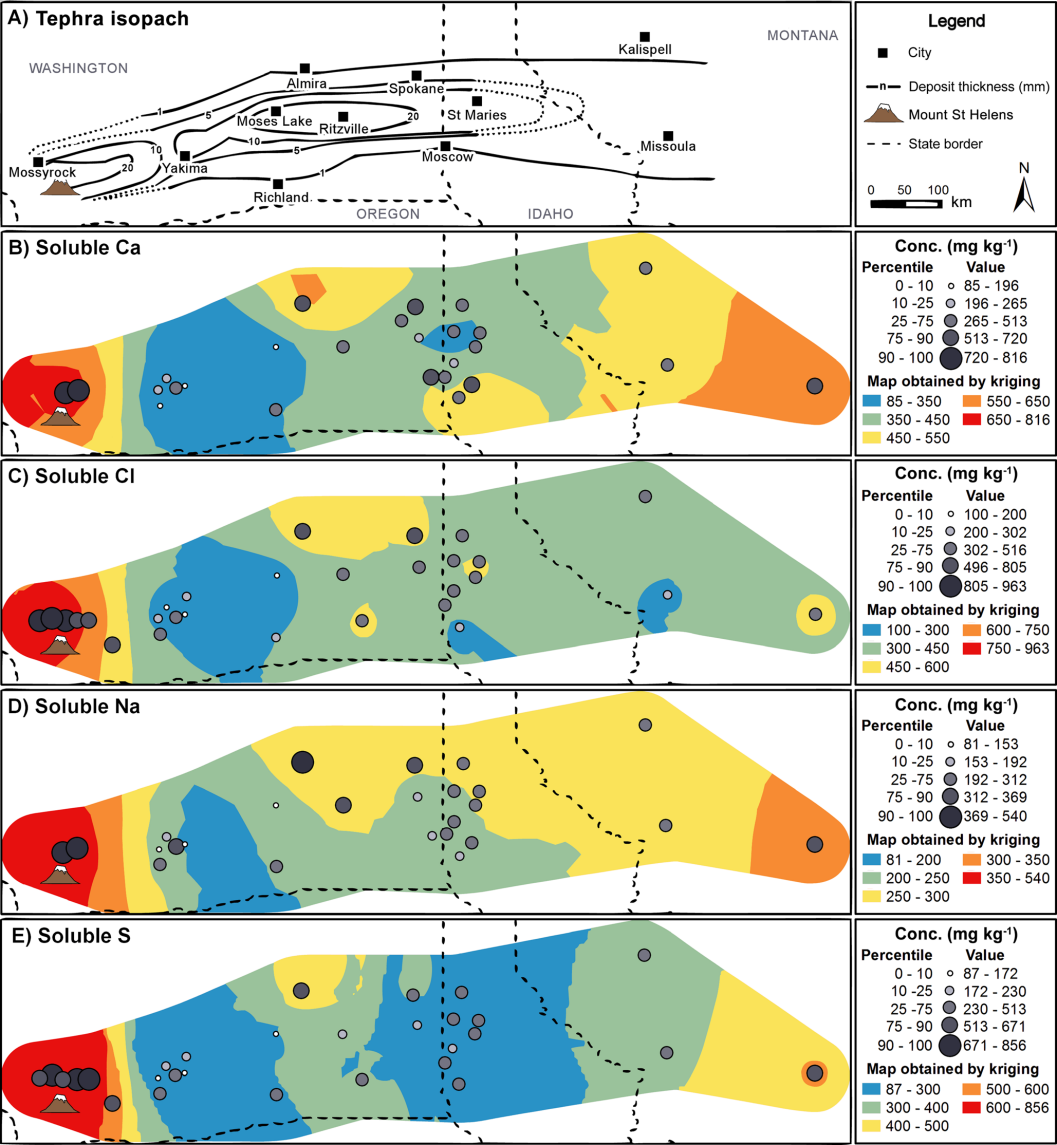
Maps of **a** MSH tephra deposit isopachs and **b** mean soluble Ca, **c** Cl, **d** Na and **e** S, expressed in milligrams per kilogram in tephra leachates in this study

There are no clear spatial patterns in the maps of soluble Ca, Cl, Na and S (Fig. 5); high and low values are frequently encountered at the same location (e.g. Yakima, WA). However, two significant observations can be made. First, mean soluble Ca, Cl, Na and S concentrations are consistently high around MSH. For Cl and S, 75 and 65 %, respectively, of values from the upper quartile range and 100 % from the 90th percentile are found in this area (Fig. 5); for Ca and Na, only two values, both in the 90th percentile, are located in the proximal region. Second, concentration values of all four elements are low at Yakima, WA and at Moses Lake, WA: at least 57 % of mean soluble Ca, Cl, Na and S from the lowest quartile range and all below the 10th percentile. In addition to these two spatial features, higher concentrations of leachates can be identified in a single sampling location east of Missoula, MT, and another at Almira, WA; however, there is insufficient data in these regions to confirm any possible spatial trends.

#### SSA_geo_

**Fig. 6:**
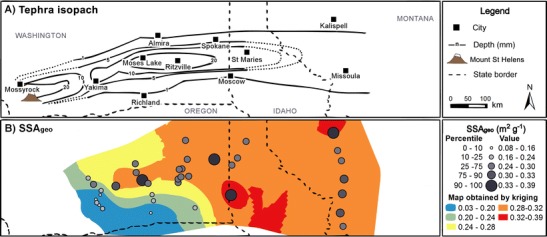
Maps of **a** MSH tephra deposit isopachs and **b** SSA_geo_ (m^2^ g^−1^) values. *Black-filled circles* in **b** indicate SSA_geo_ calculated from particle size distribution data from Durant et al. (2009) and the current study. The extent of the SSA_geo_ map is limited to areas east of Yakima, due to a lack of data in proximity to MSH

The SSA_geo_ map (Fig. 6) shows a general trend of increasing SSA_geo_ from SW to NE across the tephra deposit. The lowest values are found in proximal regions; 90 % of SSA_geo_ values below the 25th percentile are located west of Ritzville, WA, and over 90 % of all SSA_geo_ values in that area are below the 33rd percentile. In contrast, 70 % of SSA_geo_ values in the 75th percentile are found beyond the WA-ID border, whilst only 25 % of all SSA_geo_ values in that region fall below that range.

#### Bulk chemical composition

**Fig. 7:**
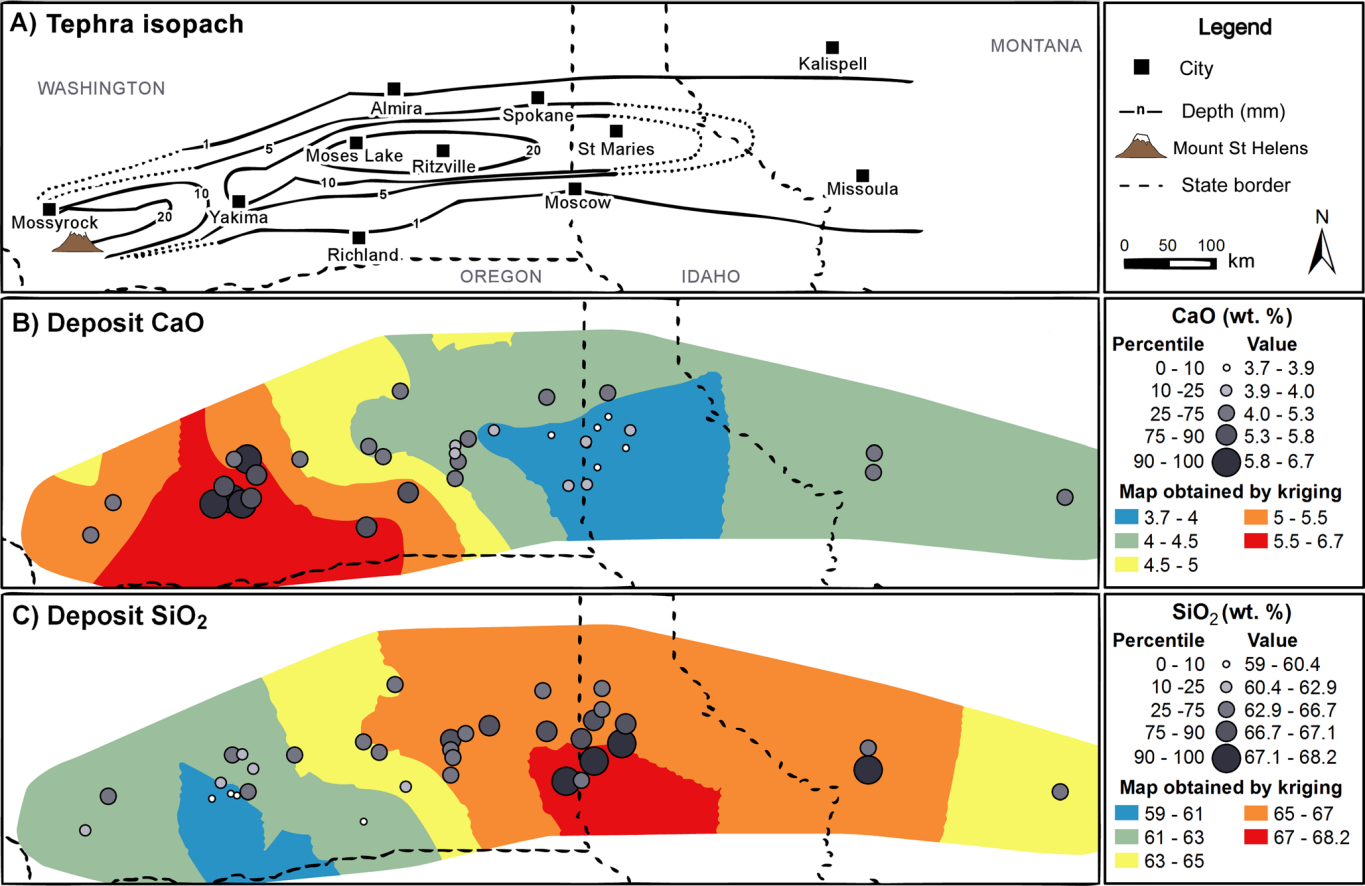
Maps of **a** MSH tephra deposit isopachs and mean deposit **b** CaO and **c**) SiO_2_ content (wt%)

Maps of tephra deposit bulk CaO and SiO_2_ content are displayed in Fig. 7. The map of tephra deposit bulk Na_2_O content is excluded (see “Validity of observed spatial trends” section). All tephra deposits with mean CaO contents within the upper quartile range are located between Yakima, WA and Ritzville, WA, whilst those in the 90th percentile are situated around Yakima itself. All mean CaO contents in the lower quartile range are located in a region bordered by Ritzville and Spokane, WA, and St. Maries and Moscow, ID (hereafter, the ‘RSSM’ region). The reverse of this trend is observed in the SiO_2_ map; over 77 % of locations reporting mean values within the lower quartile range are located between MSH and Yakima, WA, whilst over 90 % from the upper quartile range are situated in the RSSM region.

### Spatial analysis of derived data

#### SSA-normalised leachate compositions

**Fig. 8:**
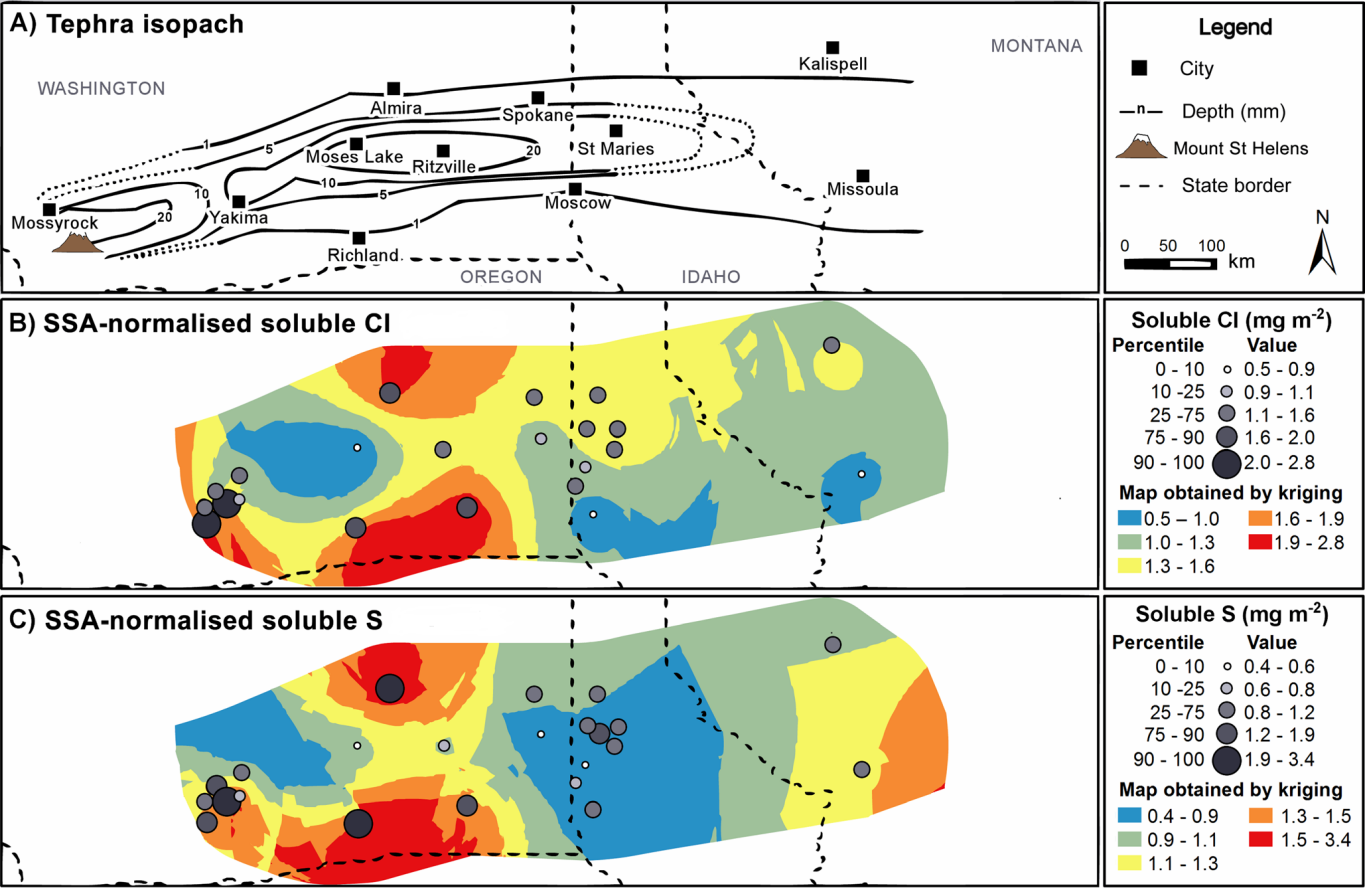
Maps of **a** MSH tephra deposits and mean soluble **b** Cl and **c** S concentrations normalised to predicted SSA_geo_ values for each sampling location, based on Fig. 4. The underlying surface maps were produced by dividing the kriging results of Cl and S by the kriging result of SSA_geo_

Soluble salts are emplaced on tephra surfaces; therefore, normalisation of leachate compositions to tephra SSA may reveal different spatial trends and features to those identified in leachate concentrations when expressed per unit mass of tephra. In Fig. 8, we illustrate this effect by normalising the soluble S and Cl concentrations (Fig. 5) at each sampling location to the SSA_geo_ values at the same location predicted by kriging (Fig. 6). The proximity of most leachate sampling locations to those sampled for tephra granulometry measurements (Fig. 1) provides confidence in the validity of the predicted SSA_geo_ values. Maps of SSA-normalised S and Cl do not exhibit clear spatial patterns. The high and low values appear to be randomly distributed on the tephra deposition zone. The only spatial feature that could be inferred is a region of low SSA-normalised soluble S between Moses Lake, WA and St Maries, ID; all samples from the lower quartile range are located in this area, with 70 % situated in the RSSM region, specifically. However, there is insufficient data, particularly between Moses Lake, WA and Ritzville, WA, to investigate this feature further.

Comparison between Figs. 5 and 8 demonstrates that normalising leachate compositions to SSA_geo_ can reveal alternative spatial trends to those identified from data expressed in concentration per unit mass of tephra; this is evidenced by the redistribution of the lowest concentrations of soluble S and Cl from the Yakima, WA area to more distal regions.

Although we attempt no interpretation of broad trends in SSA_geo_ data in proximal regions in Fig. 8, it is possible to place the leachate compositions of tephra recovered from Mossyrock and surrounding areas in context with the distal deposits by reference to stratigraphic records. Tephra within those areas predominantly featured high S and Cl concentrations per unit mass of tephra and were derived from fine-grained (∼63–125 μm) dacitic tephra deposited by the co-ignimbrite phase of the lateral blast (Hoblitt et al. 1981; Nehring and Johnston 1981). The SSA_geo_ of such fine-grained tephra deposits may thus be comparable to the distal deposits; thus, if the highest SSA_geo_ measured (0.36 m^2^ g^−1^) was assigned to leachate compositions from the Mossyrock area, all but one mean soluble Cl concentration, and two for mean soluble S, would still fall in the 90th percentiles of their respective datasets. A lower assigned SSA_geo_ would further increase these values.

#### Leachate elemental ratios

**Fig. 9:**
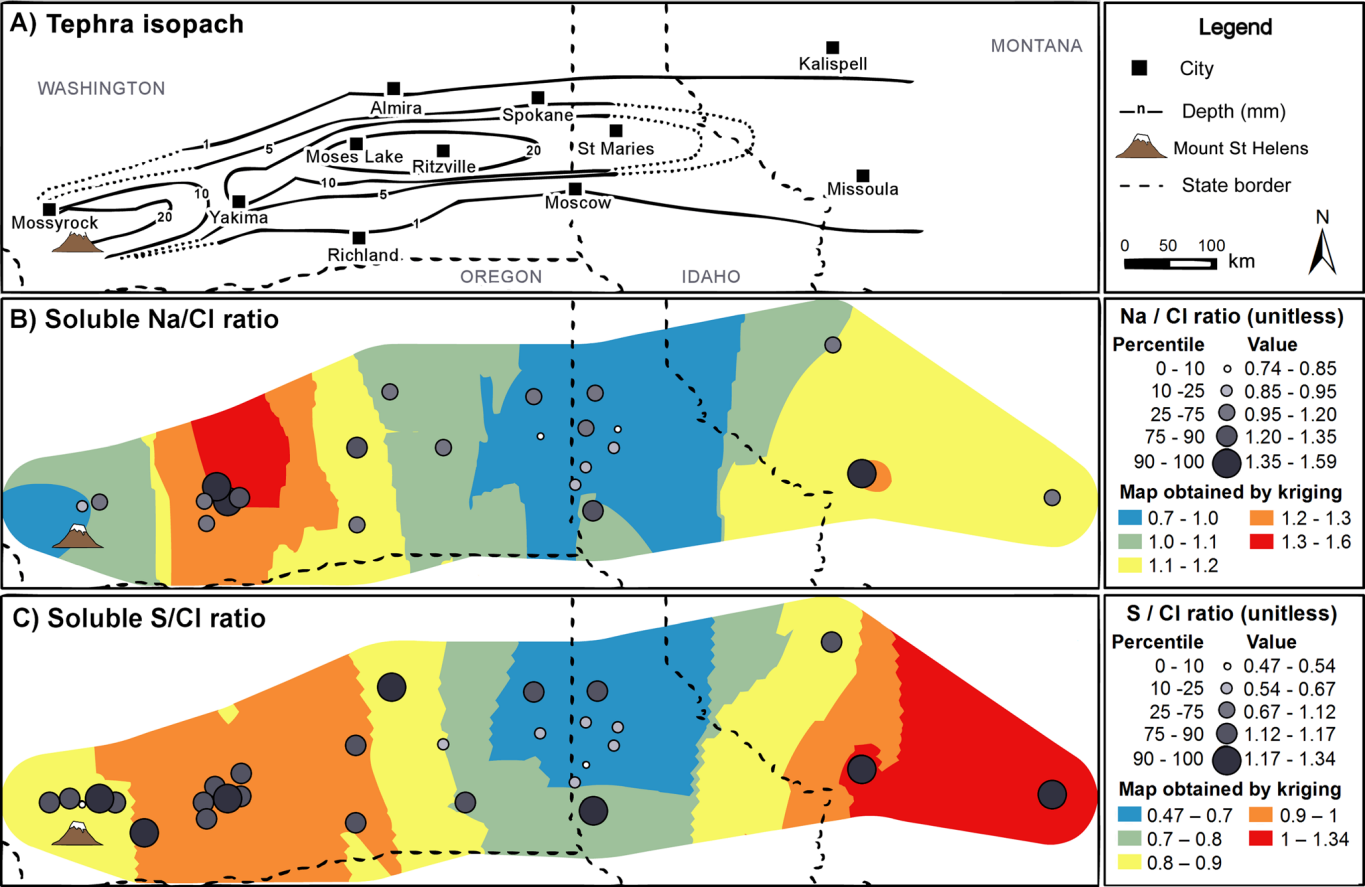
Maps of **a** MSH tephra deposits and **b** mean soluble Na/Cl and **c** S/Cl ratios in solution, based on the data from Fig. 5c–e

The maps of soluble S/Cl and Na/Cl ratios are displayed in Fig. 9, whilst the map of Ca/S is excluded (see “Validity of observed spatial trends” section). For S/Cl and Na/Cl, all but one value within the lower quartile range are located in the RSSM region. Most upper quartile range values for both Na/Cl and S/Cl ratios are located west of Yakima, WA, at the northern or southern deposit margins along the length of the distal deposits. Excluding the low Na/Cl and S/Cl region, the maps exhibit no wider spatial trends.

### Validity of observed spatial trends

#### Local variability

Mapping and interpretation of leachate and bulk chemical composition data is only appropriate if the variability of field duplicate data at individual sampling locations is less than the total variability of the dataset. If this is not the case, any inferred spatial trends could be ultimately the product of tephra sampling strategies that do not adequately represent local deposit variability, or of differences in leaching protocols. The statistical formula for pooled variance was used to calculate the variance of the field duplicate data for leachate and tephra bulk chemical composition data. Table 2 shows the relative contribution of field duplicate to total variance (%) for all raw data, i.e. soluble Ca, Cl, Na and S and bulk tephra CaO, Na_2_O and SiO_2_ content, and for derived data, i.e. soluble Ca/S, Na/Cl and S/Cl ratios. We assume that if field duplicate variance is greater than 50 % of the total variance of the whole dataset, mapping of data, and accordingly, interpretation of any spatial features, is not justified. The contribution of field duplicate variance for all datasets is less than 50 %, with the exception of Na_2_O (71 %) and Ca/S (64 %); hence, the latter two datasets were excluded in our analysis.

**Table 2:** Pooled variance of collated datasets (Tv), all field duplicate data (Fv) within those datasets and the relative contribution of Fv to Tv (%), for soluble Ca, Cl, Na and S; ratios of soluble Ca/S, Na/Cl and S/Cl; and bulk tephra CaO, Na_2_O and SiO_2_ content

Dataset	Total variance (Tv)	Field duplicate variance (Fv)	Fv/Tv (%)
Leachate			
Total conc. (mg kg^−1^)			
Ca	45,000	9530	21
Cl	45,200	14,400	32
Na	12,100	2990	25
S	42,900	7960	19
Ratio (unitless)			
Ca/S	0.0217	0.014	64
Na/Cl	0.0317	0.012	38
S/Cl	0.0654	0.0246	38
Bulk ash			
Composition (wt%)			
CaO	0.636	0.162	25
Na_2_O	0.0326	0.0233	71
SiO_2_	6.28	2.29	36

#### Influence of variable leaching times

**Fig. 10:**
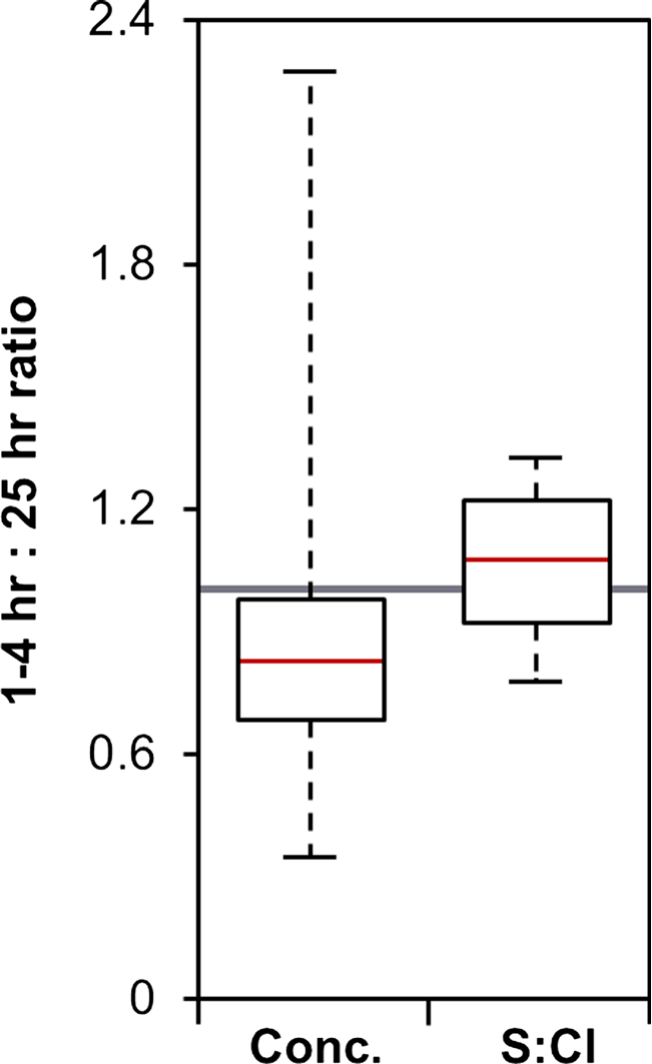
Boxplot comparison of ratios of (i) total soluble Ca, Cl, S and Na in 1–4 h relative to 25 h leaching experiments at eight locations across the MSH tephra deposit and (ii) soluble S/Cl ratios in 1–4 h relative to 25 h leaching experiments at four locations across the MSH tephra deposit. The *red line* indicates the median of the respective datasets, whilst the extent of the *white bar* covers the interquartile ranges. The extent of the *dashed lines* indicates the maximum and minimum values of those datasets

Tephra samples leached for 25 h may release proportionally more soluble Ca, Cl, Na and S than those leached for 4 h or fewer. This is demonstrated at eight locations within the collated dataset, which contain leachate compositions derived from one or more tephra samples which were leached for (i) a period of between 1 and 4 h (i.e. Fruchter et al. 1980; Hinkley et al. 1987, Nehring and Johnston 1981) and (ii) 25 h (Smith et al. 1983). At each location, we calculated the mean soluble Ca, Cl, Na and S concentrations for, and thus the mean relative difference between, leachate compositions from the two time periods. Figure 10 shows that across all four elements at the eight locations, the median ratio of 1–4:25 h data is 0.80, and all values within the interquartile range are less than unity. However, Fig. 10 also shows that there is only a weak dependence on leaching time for mean S/Cl ratios, although only four of the eight locations provide data for these calculations. These data suggest that whilst S/Cl ratios are unaffected, short duration leaching experiments underestimate the true concentrations of elements in solution. As the majority of leachate compositions in our analysis are derived from 1 to 4 h leaching experiments, we instead consider that soluble Ca, Cl, S and Na concentrations are consistently overestimated in locations where tephra samples were leached for 25 h.

## Discussion

### Syn- and post-eruptive origins of leachate spatial features

We identified two distinct deposit features: (i) high mean soluble Ca, Cl, Na and S concentrations per unit mass (Fig. 5) and per unit tephra particle surface area (Fig. 8), in fine-grained samples from blast zone deposits to the north of the volcano, and (ii) a region of low S/Cl, Na/Cl and SSA-normalised soluble S concentrations in the RSSM region (Fig. 9). The effect of leaching time does not compromise the validity of these spatial features; only one location of five in the proximal region includes data from 25 h leaching experiments, and the exclusion of 25 h data in the distal field only serves to magnify the disparity between the two regions. Similarly, the variation in S/Cl ratios observed is slight and cannot account for the observed discrepancy between low S/Cl ratios in the RSSM region and higher values in the surrounding area. We therefore interpret these identified spatial features by reference to our current understanding of gas-tephra interactions.

#### Proximal enrichments in soluble S and Cl

**Fig. 11:**
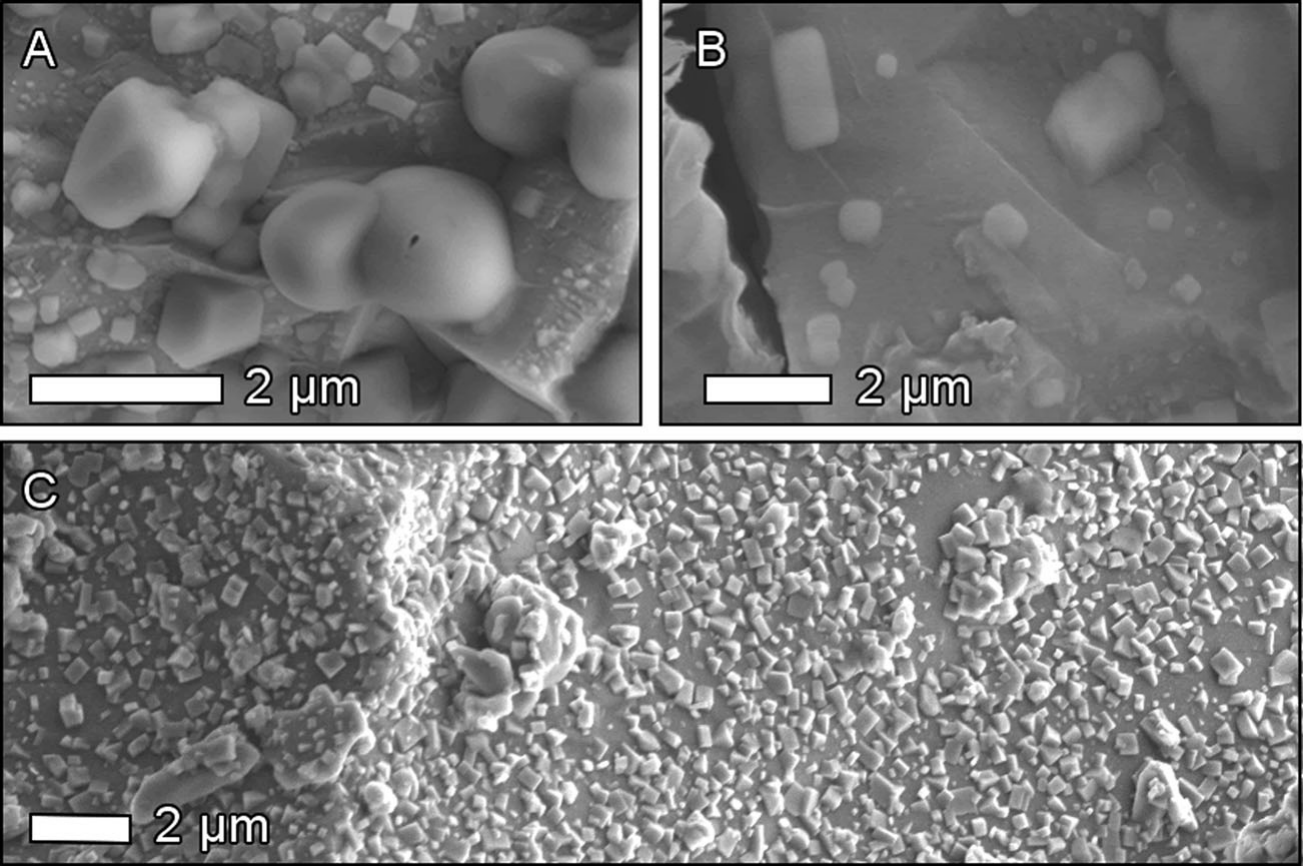
SEM images of **a** internal surface of MSH dome rock sampled directly by CVO helicopter in May 2004; **b** internal surface of 1980 cryptodome dacite boulder, recovered from Toutle River, WA in 2009; and **c** dacite glass surfaces coated with cubic CaSO_4_ deposits formed after exposure to 1 % SO_2_ in air at 800 °C for 3600 s (Ayris et al. 2013). Cubic and rectangular deposits, ranging from hundreds of nanometres to micrometres in size, are strongly indicative of the presence of soluble surface salts on these samples

The proximal enrichments in soluble S and Cl were previously noted by Stoiber et al. (1981) who suggested that this was indicative of interactions with a ‘large magmatic gas component in the directed blast and early in the eruption which decreased with time’. This gas component was considered to be SO_2_-rich and HCl-poor, in contrast to that erupted in the later stages of the eruption. However, the proximal enrichments in soluble S and Cl can also be attributed to a prolonged period of pre-eruptive gas-rock interactions within the cryptodome. Rock samples taken from the dome formed at MSH in 2004 showed extensive cubic and bleb-like surface deposits on internal surfaces (Fig. 11), morphologically identical to CaSO_4_ and NaCl deposits formed by high-temperature adsorption on volcanic glass surfaces (Ayris et al. 2013, 2014). Similar deposits were observed on internal surfaces of blast zone deposits from the MSH eruption (Fig. 11). As the cryptodome was emplaced over a period of several weeks (Cashman 1992), the timescale of pre-eruptive gas adsorption may be up to five orders of magnitude longer than during the eruption itself (e.g. Mastin 2007). Crucially, this model can account for the observed leachate feature without necessitating a change in magmatic gas composition during the eruption.

#### Distal depletions in elemental ratios and SSA-normalised S

The lowest S/Cl, Na/Cl and SSA-normalised soluble S concentrations in tephra leachates are identified in the RSSM region, although the validity of the region of low SSA-normalised soluble S, which may have its onset as far west as Moses Lake, WA, is uncertain. These features coincide with increasingly Si-rich, Ca-poor tephra deposits (Fig. 7b, c), with an apparent locus in the RSSM area. They also coincide with the distal maximum in the deposit mass accumulation, where the heaviest deposition of weakly bound tephra cluster aggregates occurred (Durant et al. 2009; Sorem 1982).

Compositional dependences on leachate chemistry across the tephra deposit have been noted by Hinkley et al. (1987); soluble S and Ca were found to be positively correlated with tephra deposit CaO content and negatively correlated with tephra deposit SiO_2_ content. Such correlations may be indicative of high-temperature adsorption of SO_2_ by tephra surfaces in the first seconds after tephra emission (Ayris et al. 2013, 2014). In short duration experiments on silicate glasses, Ayris et al. (2013) observed that high-temperature SO_2_ adsorption, forming CaSO_4_, increased with glass Ca content. That study assumed crystal phases to be unreactive to SO_2_, but Henley et al. (2015) stated that, albeit over longer timescales, crystalline and amorphous anorthite (CaAl_2_Si_2_O_8_) exhibited comparable reactivity with SO_2_. Under the assumption of the same comparable reactivity, high-temperature SO_2_ uptake, and accordingly CaSO_4_ formation, may be broadly correlated with bulk tephra CaO content, irrespective of mineralogy.

The potential for HCl adsorption within the hot eruption plume may be significantly less than that of SO_2_. Ayris et al. (2014) observed that high-temperature adsorption of HCl was negligible in dacite and rhyolite glasses, attributing this to limited reactivity of Na^+^ coordinated with tetrahedral [AlO_4_]^−^ and [FeO_4_]^−^ groups within those materials. Limited high-temperature adsorption may instead imply that HCl uptake is dominated by scavenging mechanisms acting in the cold volcanic cloud. Based on numerical simulations using the Active Tracer High Resolution Atmospheric Model (ATHAM), Textor et al. (2003) predicted that the high solubility of HCl would result in its rapid dissolution into liquid water and ice, whether as individual hydrometeors or as coatings on tephra surfaces (hydrometeor-tephra aggregates). In their simulations of a large stratospheric eruption, approximately half of all erupted HCl was sequestered into hydrometeors and hydrometeor-tephra aggregates within 60 min of the eruption onset. In contrast, virtually all SO_2_, being poorly soluble in either water or ice, remained within the volcanic cloud. As Sarna‐Wojcicki et al. (1981a) report that within 1 h of the start of the eruption, tephra deposition was confined to areas west of Yakima, it would be expected that hydrometeor-tephra aggregate scavenging of HCl would be a predominantly proximal phenomenon.

In combination with tephra dispersal, aggregation and sedimentation processes, the SO_2_ and HCl uptake models proposed can explain the observed spatial features within MSH leachate data. In the high-temperature eruption plume, SO_2_ would be most efficiently scavenged by the most Ca-rich particles; at MSH, these were dense crystal-rich tephra, more extensively produced during the early eruption of highly evolved cryptodome material than in the later eruption of juvenile magma (Sarna‐Wojcicki et al. 1981a; Scheidegger et al. 1982). In either case, these tephra were preferentially deposited in proximal regions. In contrast, Ca-poor silicic tephra with limited reactivity to SO_2_ was deposited in the RSSM region. If HCl scavenging occurs in proximal regions and is thus dictated by solubility in water and ice coatings on tephra surfaces, then there should be no spatial trend, other than a dependence on tephra SSA, and hence at least partially on granulometry, in soluble Cl concentrations across the tephra deposit. In this scenario, S/Cl ratios across the deposit would be driven by the variable reactivity of tephra to SO_2_, mediated by temporal changes within the eruption and by tephra sedimentation patterns. Such an uptake-dependent model explains the low SSA-normalised soluble S and S/Cl regions noted here and also offers an alternative explanation for the varying S/Cl ratios in ‘early’ and ‘late’ tephra noted by Stoiber et al. (1981).

The abundance of Ca-poor tephra in the RSSM region, and hence the particular spatial location of low S/Cl ratios, can be attributed to the formation and fallout of tephra aggregates. Durant et al. (2009) proposed that observations of weakly turbulent mammatus lobes at the base of the volcanic cloud over a wide area (including Ephrata, WA; Moses Lake, WA; and Vantage, WA) implicated bulk settling of the cloud layer driven by ice crystal formation and sublimation at the cloud base. Preferential aggregation of ice-laden, and hence Cl-rich, ultra-fine tephra (*p*_d_ 8–31 μm), principally comprised of silicic pumice and glass shards (Carey and Sigurdsson 1982), occurred in this region. Subsequent passage through the 0 °C isotherm caused ice to melt and form a liquid phase, which increased the rate of particle aggregation (Durant et al. 2009). Although Textor et al. (2003) predicted that HCl would be degassed during ice melting and/or sublimation, their models exclude the chemical interaction of HCl with the tephra surface. It may be possible that both prior to freezing and after thawing, acidic liquid films leach alkali and alkaline-earth cations from tephra surfaces. However, in the RSSM region, the limited capacity for leaching of Na^+^ by HCl in highly silicic glass shards (Ayris et al. 2014) may promote the additional leaching of other cations. These would be ultimately deposited as assorted chloride salts on tephra aggregate surfaces during evaporation of the newly thawed liquid film, resulting in a low Na/Cl ratio in leachate compositions, as is evident in Fig. 9.

### Implications for leachate analysis

#### Standardised analytical techniques

The 2013 IVHHN working group report ‘Protocol for analysis of volcanic ash samples for assessment of hazards from leachable elements’ (Stewart et al. 2013) offers a revised protocol of recommended practices for sample collection, storage, preparation and leaching, to promote acquisition of high-quality leachate compositions which can be more easily compared to that of other studies. Our analysis illustrates the utility of such protocols, as their use would have precluded any assumptions regarding leachate composition comparability. However, we emphasise that standardised leachate protocols do not guarantee a dataset free from analytical artefacts, and thus, should be complemented by secondary supporting analyses. In our interrogation of the MSH data, we noted that the short leaching times used in some studies, comparable to those recommended in the IVHHN guidelines, only achieved partial dissolution of soluble salts. Confidence in the representativeness of these leachates was only acquired via comparison with data derived from longer duration leaching experiments (e.g. Taylor and Lichte 1980; Smith et al. 1983; Jones and Gislason 2008). Our analysis also highlighted the possibility of systematic analytical error in the data of Stoiber et al. (1981), whereby soluble S and Cl concentrations were consistently higher than those of other studies at the same location. As systematic analytical error is difficult to detect, future leachate studies would benefit from a universal reference material, i.e. a well-characterised tephra sample with known leachate composition, verified by independent laboratories, or a synthetic tephra material which can be consistently reproduced in large quantities.

#### Spatial and temporal variability

The spatial features identified in our analysis demonstrate that small leachate datasets from large tephra deposits can fail to represent the complexities of the wider deposit. Whilst Stoiber et al. (1981) examined seven samples from Yakima, Spokane and Missoula and noted that S/Cl ratios increased with increasing distance from the volcano, our analysis identified a region of low S/Cl ratios near Spokane, east of the WA-ID border. Thus, the inferred trend of Stoiber et al. (1981) is an artefact of undersampling. However, even our collated dataset is subject to sampling density limitations; the deposit margins and most distal deposits were poorly sampled, notably in the heavily forested regions of northern Idaho (Fig. 1). This undersampling may mask unidentified spatial trends, or alter the extent, and hence interpretation, of those already identified. A more extensive leachate dataset with a homogeneous distribution of samples across the deposit would have better resolved the observed, or additional, spatial features.

Additionally, although the uptake-dependent model proposed in the section “Syn- and post-eruptive origins of leachate spatial features” offers an explanation for features noted in time-series leachate compositions (e.g. Stoiber et al. 1981; Hinkley et al. 1987), we note that such data are scarce. It is possible that with a greater quantity of similar time-series leaching, if coupled with sampling of other deposit properties (i.e. chemical composition, mineralogy) that further evidence in support of, or perhaps contrary to, the proposed model, could have been obtained. However, it is crucial to emphasise that in a time-dependent analysis, leachate datasets must still be (a) spatially representative and (b) coupled with detailed analysis of deposit stratigraphy and tephra physical and chemical properties.

In any spatio-temporal interrogation of leachate data, it is vital to consider the influence of local-scale intra-deposit variability. Field duplicate variability of total soluble Ca, Cl, S and Na concentrations per unit mass of tephra was low and may be most strongly influenced by varying SSA and the influence of different leaching times. However, the large variability of S/Cl and Na/Cl ratios (38 %, Table 2), presumed to be independent of these variables, may therefore be indicative of natural deposit variation. This possibility highlights the risk that single tephra samples may poorly represent local deposit variability in leachate compositions, requiring more extensive sampling of each location. In the current study, such data could have validated samples previously considered to be outliers, or identified additional data as anomalous. Current IVHHN guidelines recommend acquiring and compositing multiple samples from an area where deposits appear heterogeneous. For any spatial analysis of leachate compositions, greater sampling may be necessary in all cases, as there is no visible indicator of leachate heterogeneity. Similarly, whilst appropriate for impact assessment, pre-analysis compositing of samples to create a ‘blind’ mean would be undesirable for spatio-temporal or mechanistic interrogations, as it prevents any measure of local-scale variability. This could lead to over-interpretation of small variations in leachate compositions and the conflation of local and regional-scale variability.

#### Pristine tephra

**Fig. 12:**
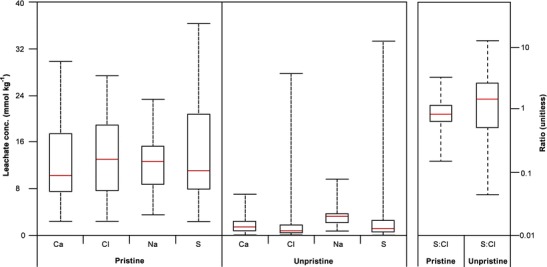
Boxplot comparison of soluble Ca, Cl, Na and S concentrations, expressed in millimoles per kilogram to permit plotting on the same scale, and S/Cl ratios, from all pristine and unpristine tephra reported in Hinkley et al. (1987) and Nehring and Johnston (1981)

It is well established that soluble salts can be dissolved by rainfall, and we accordingly excluded 49 leachate compositions from our analysis. However, the extent to which leachate compositions can be compromised by rainfall bears emphasis. At MSH, Hinkley et al. (1987) reported that tephra recovered from the Ritzville area, which received 45 mm of rain between May 18 and June 18, had lost in excess of 75 % of soluble S and Cl. We additionally compared the concentrations of soluble Ca, Cl, Na and S and the S/Cl ratios, of all 49 unpristine samples to the pristine dataset (Fig. 12). For all elements in unpristine tephra, all concentrations below the 75th percentile of their respective datasets are lower than even the lowest concentrations in the pristine dataset. Furthermore, the S/Cl ratios of unpristine samples are dissimilar to those of pristine tephra, perhaps reflecting the dissolution of S- and Cl-bearing compounds at different rates or in response to varying quantities of rainfall. Neither of these observations can be attributed to the spatial distribution of tephra samples, as the majority are recovered from areas either previously sampled, or in proximity to those areas. Thus, we emphasise that for any quantitative analysis of leachate compositions, the collection of pristine samples is of absolute importance, and echo the recommendations of the 2013 IVHHN guidelines in that researchers must ‘try to collect tephra in a pristine (dry, not rained on) condition’.

## Conclusion

We investigated the spatial structure of tephra leachate compositions from the MSH eruption, contrasting it with those of tephra deposit granulometry and chemical composition. We noted elevated leachate Ca, Cl, Na and S concentrations in blast zone deposits and a region of low S/Cl and Na/Cl ratios bounded by Ritzville and Spokane (WA) and Moscow and St. Maries (ID). Proximal enrichments may be the result of prolonged pre-eruptive exposure to magmatic gases during cryptodome growth. Conversely, the low S/Cl and Na/Cl ratios in the RSSM region may be due to the variable reactivity of ash to SO_2_ within the high-temperature eruption plume, and subsequent scavenging of HCl by ice- or water-coated ash, manifested in terrestrial deposits via both changes in magma composition and the influence of tephra dispersal and sedimentation patterns. However, analysis and mechanistic interpretation of these spatial features remains limited by the availability of samples, as well as insufficient characterisation and reporting of deposit stratigraphy and the physico-chemical properties of the tephra; thus, the spatial and temporal variability of leachate data remains poorly represented in both local areas and across the entire deposit. The influence of such limitations on our analysis emphasises the need for caution in any quantitative interrogation of leachate datasets. Additionally, future studies wishing to investigate gas-tephra interaction mechanisms, eruption dynamics or magmatic gas fluxes via leachate analysis should additionally construct and conduct their tephra sampling campaigns specifically to avoid those same limitations. Ultimately, to derive detailed mechanistic insights from leachate compositions may require sampling and characterisation of tephra deposits on an equal or greater scale than that associated with MSH. However, such an undertaking may only be possible in the event of a similar eruption, where extensive and accessible tephra deposits are emplaced within a highly populated continental landmass.
